# Preventing Respiratory Failure After Cardiac Surgery Using Post-extubation Bilevel Positive Airway Pressure Therapy

**DOI:** 10.7759/cureus.4236

**Published:** 2019-03-12

**Authors:** Nathaniel Melton, John F Lazar, William K Childers, Douglas Anderson, Nikhil P Jaik, David B Loran, Clarke Woods, Mubashir A Mumtaz

**Affiliations:** 1 General Surgery, University of Pittsburgh Medical Center Pinnacle, Harrisburg, USA; 2 Cardiovascular Thoracic Surgery, University of Pittsburgh Medical Center Pinnacle, Harrisburg, USA; 3 Miscellaneous, University of Pittsburgh Medical Center Pinnacle, Harrisburg, USA

**Keywords:** cardiac, outcomes, physiology, postoperative care, pulmonary function, respiratory therapy, ventilation

## Abstract

Objective: Our study aimed to evaluate if an extubation protocol for all post-operative cardiac patients in the cardiothoracic intensive care unit using intermittent bilevel positive airway pressure (BiPAP) could reduce the rate of re-intubation.

Methods: A total of 1,718 patients undergoing cardiac surgery from May 2012 to April 2016 were analyzed. Patients from May 2014 to April 2016 were included in a post-extubation BiPAP therapy protocol that included one hour of BiPAP followed by three hours of a nasal cannula for 24 hours after extubation in the cardiothoracic intensive care unit. The protocol cohort was retrospectively compared to a control group (nasal cannula only) from May 2012 to April 2014. All demographic and outcome data were analyzed from our institution’s Society of Thoracic Surgeons (STS) Cardiac Database.

Results: There was no statistical difference in the rate of re-intubation between the BiPAP group (n = 35; 4.07%) and the control group (*n* = 34; 3.96%; *p* = 0.9022). Sub-group analysis of the 69 re-intubated patients identified several significant risk factors: prior valve surgery (*p* = 0.028), chronic lung disease (*p* = 0.0343), emergent operation (*p* = 0.0016), longer operating room time (*p* = 0.0109), cardiopulmonary bypass time (*p* = 0.0086), higher STS predicted risk of mortality score (*p* = 0.0015). Re-intubation was associated with higher 30-day mortality rates (*p* = 0.0026), prolonged cardiothoracic intensive care unit length of stay (*p* < 0.0001), and hospital length of stay (*p* < 0.0001).

Conclusion: While a BiPAP protocol did not show a significant difference in re-intubation rates after cardiac surgery, the subgroup analysis of re-intubated patients showed several significant risk factors for re-intubation. Early identification of these risk factors when considering extubation may help teams avoid associated morbidity and mortality outcomes.

## Introduction

Acute respiratory failure and pulmonary dysfunction is a significant cause of morbidity and mortality following cardiac surgery [1-2]. This pulmonary dysfunction stems from impairment of gas exchange and lung mechanics secondary to multiple factors including use of cardiopulmonary bypass, activation of the inflammatory cascade, atelectasis, decreased thoracic compliance, pleural effusions, postoperative pain, and diaphragmatic dysfunction. Non-invasive positive pressure ventilation (NPPV) is often used to correct postoperative pulmonary dysfunction and to treat respiratory failure in the intensive care unit (ICU). Several studies do support the use of NPPV to treat postoperative respiratory failure after non-cardiac surgery [3-6].

Bilevel positive airway pressure (BiPAP) is a commonly used form of NPPV that uses higher pressures during inspiration compared to expiration. Many studies have looked at different methods of NPPV, including BiPAP to prevent and treat respiratory failure following cardiac surgery [7-9]. These studies have shown that BiPAP can improve hypoxemia and decrease atelectasis after extubation following cardiac surgery [7-10]. However, there is not much evidence on the use of BiPAP in preventing re-intubation following cardiac surgery [8,11].

This observational study aimed to evaluate if the initiation of an extubation protocol for all post-operative cardiac patients in the cardiothoracic intensive care unit (CTICU) using intermittent BiPAP could reduce the rate of re-intubation. We hypothesized that our rates of re-intubation would decrease following the implementation of our BiPAP protocol. Secondarily, we sought to identify factors demonstrating increased risk for re-intubation following cardiac surgery.

## Materials and methods

A post-extubation BiPAP therapy protocol was designed by a multidisciplinary team of cardiac surgeons, respiratory therapists, and medical intensivists at our institution and was subsequently implemented in May 2014. Our protocol, which was applied to all post-cardiac surgery patients, consisted of one hour of BiPAP therapy, followed by three hours of a nasal cannula for 24 hours starting half an hour after extubation in all patients post-cardiac surgery. Patients also used incentive spirometry and positive end-expiratory pressure (PEEP) therapy every two hours. If BiPAP could not be tolerated, the patient was placed on high flow nasal oxygen.

After obtaining approval of our institutional review board and waiving patient consent, a total of 1,718 patients undergoing cardiac surgery from May 2012 to April 2016 were retrospectively analyzed. Eight hundred and fifty-nine patients from May 2014 to April 2016 were in the protocol cohort. This protocol cohort was retrospectively compared to the control group (nasal cannula only), of 859 patients, from May 2012 to April 2014.

Our institution’s Society of Thoracic Surgeons Cardiac Database was used to compare demographic and outcome data between the cohorts. The primary outcome was the percent of patients re-intubated. Secondary outcomes included postoperative pneumonia, 30-day mortality, 30-day re-admissions, and length of stay. A subgroup analysis of re-intubated patients was performed to identify factors that were associated with an increased risk for reintubation following cardiac surgery. We reported continuous variables as mean and/or median, standard deviation and range and categorical variables as number (percent). We used Student’s *t*-test to analyze between-group differences for the continuous variables. When it was determined that variances for the comparisons of continuous data were unequal, Welch-Satterthwaite t-test statistics were used. We used a chi-square test to analyze between-group differences for the categorical variables. The Fisher's exact test was employed when any of the expected frequencies was five or less. All the analyses were done by SAS ver 9.4 (SAS Institute, Cary, NC). A *p*-value of <0.05 was considered significant.

## Results

A total of 1,718 patients that underwent cardiac surgery from May 2012 to April 2016 were analyzed. Eight hundred and fifty-nine patients were equally divided into a BiPAP protocol cohort and control cohort and were compared. The most common surgery among both groups was coronary artery bypass and was performed significantly more in the BiPAP group (48.31% vs. 40.63%, *p* = 0.0014). Aortic valve replacement was performed significantly more in the control group (21.65% vs. 11.18%, *p* < 0.0001).

We compared non-respiratory and respiratory demographics, operative characteristics, and postoperative complications between the two groups. Among the non-respiratory demographics, the BiPAP group had a significantly greater number of patients with cerebrovascular disease (22.82% vs. 13.39%, *p* < 0.0001), history of heart failure (33.18% vs. 21.65%, *p* < 0.0001), history of percutaneous coronary intervention (PCI; 23.05% vs. 18.74%, *p* = 0.0281), and history of coronary stenting (20.84% vs. 17.11%, *p* = 0.0490). As for the respiratory demographics, the BiPAP group had significantly more former smokers (42.03% vs. 9.66%, *p* < 0.0001), more patients using daily bronchodilators (15.72% vs. 12.11%, *p* < 0.0307), and patients with a history of pneumonia (10.36% vs. 6.87%, *p* < 0.0099). Pulmonary function tests were performed on 95.93% of the patients in the BiPAP group, compared to 94.88% in the control group (*p* = 0.2999). The control group did have significantly more patients with a forced expiratory volume in one second (FEV1) <60% (15.60% vs. 11.53%, *p* < 0.0103; Table 1).

**Table 1:** Patient characteristics BiPAP, bilevel positive airway pressure; PFT, pulmonary function test; FEV_1_,_ _forced expiratory volume in one second

	BiPAP Group (n = 859)	Control Group (n = 859)	p-Value
Patient demographics			
Age in years – median	68	69	0.7238
Sex (Male) – number (%)	591 (68.80%)	569 (66.24%)	0.2570
Patient medical history – number (%)			
Diabetes	321 (37.37%)	319 (37.14%)	0.9205
Hypertension	784 (91.27%)	762 (88.71%)	0.0770
Hyperlipidemia	726 (84.52%)	719 (83.70%)	0.6441
Renal failure – dialysis	18 (2.10%)	28 (3.26%)	0.1350
Cerebrovascular disease	196 (22.82%)	115 (13.39%)	<0.0001
Prior myocardial infarction	291 (33.88%)	261 (30.38%)	0.1212
Prior coronary artery bypass	41 (4.77%)	57 (6.64%)	0.0960
Prior valve	46 (5.36%)	46 (5.36%)	1.0000
History of heart failure	285 (33.18%)	186 (21.65%)	<0.0001
History of percutaneous coronary intervention	198 (23.05%)	161 (18.74%)	0.0281
History of coronary stenting	179 (20.84%)	147 (17.11%)	0.0490
Former smoker	361 (42.03%)	83 (9.66%)	<0.0001
Current smoker	112 (13.04%)	107 (12.46%)	0.7176
Chronic lung disease	289 (33.64%)	307 (35.74%)	0.3616
PFT	824 (95.93%)	815 (94.88%)	0.2999
FEV_1_ <60%	99 (11.53%)	134 (15.60%)	0.0103
FEV_1_ >80%	476 (55.41%)	442 (51.46%)	0.1509
FEV_1_ (median)	83%	81%	0.0080
Home oxygen	13 (1.51%)	15 (1.75%)	0.7031
Bronchodilator therapy	135 (15.72%)	104 (12.11%)	0.0307
History of pneumonia	89 (10.36%)	59 (6.87%)	0.0099

Patients in the BiPAP group were more likely to undergo elective surgery (73.92% vs. 64.24%, *p* < 0.0001), while the control group underwent more urgent cases (34.11% vs. 23.98%, *p* < 0.0001). Patients in the BiPAP group spent longer time in the operating room (4.28 vs. 3.97 hours, *p* < 0.0001) and on cardiopulmonary bypass (75 vs. 69 minutes, *p* = 0.0024). In the BiPAP group, patients were more likely to be extubated within six hours after case completion (53.32% vs. 35.97%, *p* < 0.0001), while the control group significantly more patients being extubated greater than 12 hours after case completion (27.47% vs. 15.60%, *p* < 0.0001). More patients in the control group were extubated in the operating room (12.46% vs. 6.64%, *p* < 0.0001). Patients in the control group had a higher incidence of pleural effusion (3.14% vs. 0.35%, *p* < 0.0001) requiring tube thoracostomy, while the BiPAP group had higher incidence of pneumothorax (0.70% vs. 0.00%, *p* = 0.0310) requiring tube thoracostomy. The control group had more patients develop postoperative atrial fibrillation (27.36% vs. 21.89%, *p* = 0.0099; Table 2). 

**Table 2:** Operative characteristics OR, operating room; BiPAP, bilevel positive airway pressure

	BiPAP Group (n = 859)	Control Group (n = 859)	p-Value
Procedure type			
Elective	635 (73.92%)	551 (64.14%)	<0.0001
Urgent	206 (23.98%)	293 (34.11%)	<0.0001
Emergent	18 (2.10%)	15 (1.75%)	0.5980
OR time			
Time in OR (hours) – median	4.28	3.97	<0.0001
Time in OR <4 hours – no.%	331 (38.53%)	441 (51.34%)	<0.0001
Time in OR 4-6 hours – no.%	430 (50.06%)	376 (43.77%)	0.0090
Time in OR >6 hours – no.%	101 (11.76%)	42 (4.89%)	<0.0001
Bypass length (minutes) – median	75	69	0.0024
Intraoperative blood products – total number	81 (9.43%)	229 (26.66%)	<0.0001
Time to extubation			
Time to extubation (0-6 hours)	458 (53.32%)	309 (35.97%)	<0.0001
Time to extubation (6-9 hours)	151 (17.58%)	135 (15.72%)	0.2907
Time to extubation (9-12 hours)	65 (7.57%)	79 (9.20%)	0.2286
Time to extubation (>12 hours)	134 (15.60%)	236 (27.47%)	<0.0001
Extubated in OR	57 (6.64%)	107 (12.46%)	<0.0001
First cardiac surgery	788 (91.73%)	769 (89.52%)	0.0990
First re-do cardiac surgery	73 (8.50%)	94 (10.94%)	0.0903
STS predicted risk of mortality score – mean	0.0157	0.0175	0.0684

Our primary outcome was the number of re-intubations between the two cohorts. There was no statistical difference in the rate of re-intubation (*p* = 0.9022) between the BiPAP group (*n *= 35; 4.07%) and the control group (*n *= 34; 3.96%). For our secondary outcomes, there was no statistical difference between the BiPAP and control groups when comparing post-operative pneumonia (1.75% vs. 1.86%, *p* = 0.8562), 30-day re-admissions (6.87% vs. 8.15%, *p* = 0.3139), 30-day mortality (1.16% vs. 1.75%, *p* = 0.3138). However, the BiPAP group did have a significantly shorter total intubation time (5.4 vs. 7.4 hours, *p* < 0.0001) and length of stay (LOS; 7.6 vs. 8.6 days, *p* < 0.0001; Table 3).

**Table 3:** Study outcomes LOS, length of stay; BiPAP, bilevel positive airway pressure

	BiPAP Group (n = 859)	Control Group (n = 859)	p-Value
Re-intubated	35 (4.07%)	34 (3.96%)	0.9022
Post-operative pneumonia	15 (1.75%)	16 (1.86%)	0.8562
30-day readmissions	59 (6.87%)	70 (8.15%)	0.3139
30-day mortality	10 (1.16%)	15 (1.75%)	0.3138
Total intubation time (hours) – median	5.4	7.4	<0.0001
Total LOS (days) – mean	7.6	8.6	<0.0001

Sub-group analysis of the 69 patients re-intubated did identify several significant overall risk factors for re-intubation. These included: prior valve surgery; 8 (11.59%) vs 84 (5.09%; *p* = 0.028), chronic lung disease; 9 (13.04%) vs 97 (5.88%; *p* = 0.0343), emergent operation; 6 (8.70%) vs 27 (1.64%; *p* = 0.0016), longer OR time; 4.55 vs 4.1 hours (*p* = 0.0109), total intubation time; 8.68 vs 6.04 hours (*p* = 0.0039); cardiopulmonary bypass time; 84 versus 72 minutes (*p* = 0.0086), and higher predicted Society of Thoracic Surgeons (STS) risk of mortality score; 2.4% vs 1.6% (*p* = 0.0015). Re-intubation was associated with higher 30-day mortality rates; 5 (7.25%) vs 20 (1.21%) (p = 0.0026), prolonged CTICU LOS; 213 vs 50 hours (*p* < 0.0001), hospital LOS; 7 vs 10 days (*p* < 0.0001). In addition, patients that were re-intubated had higher incidence of: sepsis; 6 (8.70%) vs 3 (0.18%; *p* < 0.0001), stroke; 4 (5.80%) vs 17 (1.03%; p = 0.0085), pneumonia; 12 (17.39%) vs 19 (1.15%) (p < 0.0001), pleural effusion; 6 (8.70%) vs 24 (1.46%; *p* = 0.0009), pneumothorax; 2 (2.90%) vs 1 (0.06%; *p* = 0.0215), renal failure; 8 (11.59%) vs 18 (1.09%; *p* < 0.0001), pacemaker; 9 (13.04%) vs 106 (6.43%; *p* = 0.0445), cardiac arrest; 7 (10.14%) vs 8 (0.49%; *p* < 0.0001), and atrial fibrillation; 30 (43.48%) vs 392 (23.77%; *p* = 0.0002; Table 4).

**Table 4:** Significant risk factors for re-intubation and complications following re-intubation

	Reintubated (n = 69)		Not Reintubated (n = 1649)			p-Value
Age in years- mean (SD), range	69.2 (9.8)	44-86		67.5 (11.03)	21-94	0.2020
Gender (male) – number (%)	44 (63.77%)			1116 (67.68%)		0.4969
Risk Factors for Re-intubation						
Prior valve surgery	8 (11.59%)			84 (5.09%)		0.028
Congestive heart failure	26 (37.68%)			445 (26.99%)		0.051
Chronic lung disease	9 (13.04%)			97 (5.88%)		0.034
Emergent case status	6 (8.70%)			27 (1.64%)		0.0016
Time in operating room (hours) - median	4.55			4.10		0.0109
STS predicted risk of mortality score - median	2.40%			1.60%		0.0015
Complications						
Sepsis	6 (8.70%)			3 (0.18%)		<0.0001
Stroke	4 (5.80%)			17 (1.03%)		0.0085
Pneumonia	12 (17.39%)			19 (1.15%)		<0.0001
Pleural effusion (requiring intervention)	6 (8.70%)			24 (1.46%)		0.0009
Pneumothorax (requiring intervention)	2 (2.90%)			1 (0.06%)		0.0215
Renal failure	8 (11.59%)			18 (1.09%)		<0.0001
Pacemaker	9 (13.04%)			106 (6.43%)		0.0445
Cardiac arrest	7 (10.14%)			8 (0.49%)		0.0002
30-day mortality	5 (7.25%)			20 (1.21%)		0.0026
Total ICU LOS (hours)-median	213			50		<0.0001
Total LOS (days)-median	15			7		<0.0001

## Discussion

Pulmonary dysfunction is a common cause of morbidity following cardiac surgery [1-2,12]. Given the known detrimental effects this has on postoperative outcomes [1-2,12], it is imperative that respiratory failure is treated early to prevent reintubation. NPPV is used successfully to treat the respiratory failure of various etiologies, as well as, postoperative respiratory failure following non-cardiac surgery [3-6]. There is also some data to suggest that NPPV can be used to treat respiratory failure following cardiac surgery [8-9,13]. BiPAP is safe, and improves hypoxemia and decreases atelectasis following cardiac surgery [7-10]. However, there is limited data to suggest that BiPAP can be used to decrease reintubation rates following cardiac surgery [8,11].

In 2014, a combination of cardiac surgeons, respiratory therapists, and medical intensivists at our institution developed a BiPAP therapy protocol that was instituted once a patient was extubated following every cardiac surgery. This protocol placed patients on BiPAP for one hour and then a nasal cannula for three hours, starting half an hour after extubation. This cycle is repeated for 24 hours. This protocol was devised to reduce the reintubation rates for post-cardiac surgery patients. Also, it was thought that by developing a specific protocol, our nursing and respiratory therapy staff would have a simplified and structured outline for post-extubation respiratory management. Our protocol was developed after reviewing numerous studies, including a study from Celebi et al. that showed that following cardiac surgery, post-extubation hypoxemia, and atelectasis were both improved with intermittent BiPAP therapy [8].

The primary outcome of our study failed to show a difference in reintubation rates after the establishment of post-extubation intermittent BiPAP therapy protocol following cardiac surgery. This is consistent with Sagiroglu et al., who showed the early and prophylactic use of BiPAP without acute respiratory failure in the early post-extubation period following cardiac surgery did not improve reintubation rates [11]. We identified several possible key factors for the lack of significance: (a) the BiPAP population had more co-morbidities including cerebrovascular disease, prior heart failure, prior coronary intervention, former smokers, history of pneumonia, and use of daily bronchodilators; (b) It is also possible that the patients in the BiPAP arm were getting extubated too early. The STS uses time-to-extubation less than six hours following surgery as the benchmark for early extubation, which may contribute to pushing patients to be extubated within this time frame. Our study showed significantly more patients in the BiPAP group getting extubated within this 6-hour mark. However, Crawford et al. showed that operative mortality, major morbidity, and prolonged LOS did not increase until time-to-extubation reached at least 12 hours [14]. The pressure to extubate within six hours may lead to early extubation of some patients; (c) Lastly, are some factors more important than simply increasing oxygen levels and decreasing atelectasis in preventing reintubation? Our study showed that longer operating room and bypass times are associated with an increased risk of reintubation. So, it is possible that these factors and others are more important than increasing oxygen levels and decreasing atelectasis with BiPAP for preventing reintubation.

As for our secondary outcomes, which looked at identifying risk factors for re-intubation post-cardiac surgery, there were no significant differences between the cohorts when comparing post-operative pneumonia, 30-day readmissions, and 30-day mortality. The BiPAP group did have a significantly shorter total intubation time and total hospital LOS.

Several significant patient risk factors for re-intubation were identified as part of our study. These included prior valve surgery, congestive heart failure, and chronic lung disease. Our study also showed that emergent case status, longer operating room time, longer cardiopulmonary bypass times, and higher STS risk of mortality scores were associated with an increased risk of re-intubation. These risk factors are consistent with prior studies [15-16]. Going forward, these risk factors should be kept in mind when deciding to extubate the patient. These specific patients are the population that may benefit from intermittent BiPAP therapy following extubation. However, further research is needed.

Our study has several limitations. First, our study did not consider how many patients did not complete the 24-hour protocol because they could not tolerate BiPAP. Many patients in our study found BiPAP uncomfortable and could not tolerate it. Past studies have looked at using high-flow nasal oxygen instead of BiPAP to improve oxygenation after extubation following cardiac and non-cardiac surgery [7,17]. These studies showed that among cardiac and non-cardiac surgery patients with or at-risk for respiratory failure, the use of high-flow nasal oxygen therapy compared with intermittent BiPAP did not result in a higher treatment failure rate. The patients kept on BiPAP because of worsening respiratory status were excluded. The specific reason for reintubation was not documented, and therefore could not be evaluated. Lastly, this is an observational study and used historical data for our control group.

Going forward, several factors should be considered. One, the reason for re-intubation should be documented and form a part of the protocol. There are many reasons for re-intubation including failure to clear secretions, weakness, altered mental status, smoking, etc. Acute respiratory failure can be further characterized by the cause such as due to hypoxia, hypercapnia, or a combination of both. Depending on the underlying cause and type of respiratory failure, BiPAP may not affect the underlying pathophysiologic process. Also, arterial blood gas data, as well as the STS risk of re-intubation score should be documented for each patient and could be used to further characterize patients at higher risk for re-intubation who could benefit from our protocol.

 While an intermittent BiPAP protocol did not show a significant difference in re-intubation rates after cardiac surgery versus nasal cannula, subgroup analysis of re-intubated patients showed significant risk factors for re-intubation. These risk factors should be kept in mind by teams when considering post-operative extubation to avoid the associated detrimental outcomes.

## Conclusions

While a BiPAP protocol did not show a significant difference in re-intubation rates after cardiac surgery, the subgroup analysis of re-intubated patients showed several significant risk factors for re-intubation. Early identification of these risk factors when considering extubation may help teams avoid associated morbidity and mortality outcomes.
